# A guide to automated apoptosis detection: How to make sense of imaging flow cytometry data

**DOI:** 10.1371/journal.pone.0197208

**Published:** 2018-05-16

**Authors:** Dennis Pischel, Jörn H. Buchbinder, Kai Sundmacher, Inna N. Lavrik, Robert J. Flassig

**Affiliations:** 1 Process Systems Engineering, Otto-von-Guericke-University Magdeburg, Magdeburg, Germany; 2 Translational Inflammation Research, Otto-von-Guericke-University Magdeburg, Magdeburg, Germany; 3 Process Systems Engineering, Max Planck Institute for Dynamics of Complex Technical Systems, Magdeburg, Germany; Wayne State University, UNITED STATES

## Abstract

Imaging flow cytometry is a powerful experimental technique combining the strength of microscopy and flow cytometry to enable high-throughput characterization of cell populations on a detailed microscopic scale. This approach has an increasing importance for distinguishing between different cellular phenotypes such as proliferation, cell division and cell death. In the course of undergoing these different pathways, each cell is characterized by a high amount of properties. This makes it hard to filter the most relevant information for cell state discrimination. The traditional methods for cell state discrimination rely on dye based two-dimensional gating strategies ignoring information that is hidden in the high-dimensional property space. In order to make use of the information ignored by the traditional methods, we present a simple and efficient approach to distinguish biological states within a cell population based on machine learning techniques. We demonstrate the advantages and drawbacks of filter techniques combined with different classification schemes. These techniques are illustrated with two case studies of apoptosis detection in HeLa cells. Thereby we highlight (*i*) the aptitude of imaging flow cytometry regarding automated, label-free cell state discrimination and (*ii*) pitfalls that are frequently encountered. Additionally a MATLAB script is provided, which gives further insight regarding the computational work presented in this study.

## Introduction

The individual character of single cells plays an important role in defining the observed phenotype of a biological system. Cells within a population differ in all kinds of attributes, such as morphology, cell cycle state or protein abundances. To monitor the behavior of individual cells within a population and identify cell state changes, such as cell death, single cell measurement techniques have to be applied [1]. In this study, we focus on imaging flow cytometry (IFC), which allows to observe cellular structures on a microscopic scale in a high-throughput manner [2]. IFC takes single cell images within a cell population *via* bright field imaging in combination with the detection of images in different fluorescence channels. From the images, an enormous amount of information can be extracted, which results in the characterization of single cells by various properties. The traditional approach to filter the most significant information is by manual one- or two-dimensional gating. Since only low-dimensional projections are considered, this approach ignores further information hidden in the high-dimensional property space. Additionally, the properties selected for discrimination and the boundary that separates subpopulations corresponding to different phenotypes are strongly dependent on subjective expert knowledge [3, 4].

**Fig 1:**
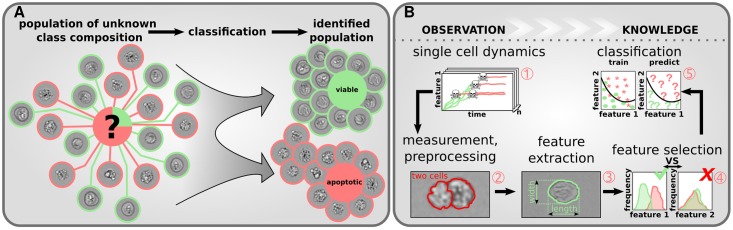
Cell state classification for imaging flow cytometry. (A) Machine learning is used to predict the unknown composition of cellular populations (*e.g.* viable (green) and apoptotic (red)). (B) The combination of experimental expertise (1, 2, 3) and data mining (3, 4, 5) facilitates the derivation of useful information from huge amounts of biological data.

To overcome these shortcomings, we present a simple tutorial on how to efficiently find discriminative information in a big data property space using machine learning. Machine learning techniques are rational methods to assign unseen objects or samples with certain properties to categorical classes [5]. The assignment follows algorithmic rules taking solely object properties into account. In this work’s case studies we consider a heterogeneous cell population consisting of several subpopulations. Each subpopulation is characterized by a specific phenotype comprising the specific class. The aim is to correctly predict the class or phenotype of so far unseen cells based on the properties obtained by IFC measurements, see Fig 1A.

The scope of this manuscript is to guide the reader through the complex process of automated cell state discrimination. Therefore, we show a generally applicable, modular workflow focussing on the computational aspects. We start with a brief review of existing machine learning algorithms, how to validate them and a short overview of filtering techniques to select promising properties for classification. In addition, these techniques are applied to two examples of apoptosis detection in HeLa cells overexpressing the death receptor CD95 (HeLa-CD95 cells) to illustrate the proposed methodology and discuss potential pitfalls.

## From measurement to classification

The process from measurement to successful cell state classification is a complex interplay of experimental know-how combined with data mining approaches, see Fig 1B. IFC allows high-throughput measurements in form of single cell images, which are corrupted by measurement noise and artifacts. By proper preprocessing, images unsuitable for analysis, *e.g.* due to pictures of overlapping cells, can be excluded. The preprocessed images are suitable for extraction of properties describing each single cell by numeric values. These properties represent physically measurable quantities and are known as features. The amount of features for IFC measurements is immense (order of 10^2^) compared to ordinary flow cytometry. Not only (*i*) morphological features, such as the size of the nucleus, but also (*ii*) spectral features, like dye specific fluorescence intensities, (*iii*) the combination of morphological and spectral features, such as translocation of dyes or (*iv*) other abstract features are available [2]. Although the interpretation of abstract features is not straightforward in biological terms, they might contain valuable information and should not be dismissed [6–9]. Due to the high-dimensional feature space and large sample size many features are correlated, redundant and may differ in information content [10]. Naturally the question arises how to select the most important features to defy the curse of dimensionality and optimize the classification performance.

Several selection approaches have been proposed including filter, wrapper and embedded techniques [11]. Filter methods are independent of the classification scheme and select features only by evaluating the intrinsic characteristics of the data. On the contrary, wrapper methods use the classification accuracy to determine the usefulness of feature subsets and embedded techniques make use of a classification scheme with integrated feature selection. Since wrapper and embedded techniques are computational very intense and prone to overfitting, we focus in this study on filtering, which stands out due to its simplicity and efficiency while yielding excellent results [12]. Especially the low computational demand and scalability to large data sets make it an ideal candidate for the analysis of IFC measurements.

For cell discrimination an algorithmic classification scheme has to be trained using data with known classes. In general, classifiers depend on several hyperparameters, which have to be tuned. Thus, a set of competing models is on-hand, whereof the best is obtained by optimization. In order to select the model which discriminates the classes best classification accuracy, robustness against data perturbation and generalization to data not used for training is considered [13]. The selected model can then be used to predict the class of new data in an automated manner.

## Feature selection *via* filtering

The aim of feature selection is to identify a subset of features for optimal classification [11, 12]. In this case, feature selection can be understood as a mapping *h* from the *n*-dimensional feature space containing all available features to an *n*_*FS*_-dimensional subspace $h:R^{n}\to R^{n_{FS}}$ with $R^{n_{FS}}\subseteq R^{n}$. The variable *n*_*FS*_ denotes the number of features obtained by feature selection. In order to arrange the features according to their information content, a ranking *via* a scoring scheme is performed. The first *n*_*FS*_ features, which achieve the highest scores, will be selected and used for further analysis.

Regarding the scoring scheme, filtering can be sectioned into univariate and multivariate techniques [11, 12]. Univariate filter algorithms do not consider feature interactions and assign each feature a score based solely on its correlation to the different classes. Thus, univariate filters are able to distinguish relevant and irrelevant features. In contrast, multivariate techniques additionally capture feature interactions, such as redundancy, which is computationally more intense.

In the following we consider data given as matrix $X\in R^{m\times n}$ with *m* denoting the number of observed samples and *n* the number of features. Further, we introduce the random variables ***x***, which represents a vector comprising all features, to describe a certain sample and the random variable *χ*_*i*_ denoting the *i*th feature. For several samples a vector of class labels ***y*** exists, which assigns one of the *n*_*c*_ distinct classes $c=\{c_{1},...,c_{n_{c}}\}$ to them.

### Mutual information maximization

Mutual information maximization (MIM) [14, 15] is a univariate feature selection approach, which uses mutual information *I* between the different classes and a certain feature *χ*_*i*_ as a measure of correlation to assign every feature a score for ranking

$$\begin{array}{c} I\left(\chi_{i},c\right)=\sum_{j}\sum_{k}P\left(\chi_{i,j},c_{k}\right)\text{log}(\frac{P(\chi_{i,j},c_{k})}{P\left(\chi_{i,j}\right)P\left(c_{k}\right)}). \end{array}$$ (1)

**Fig 2:**
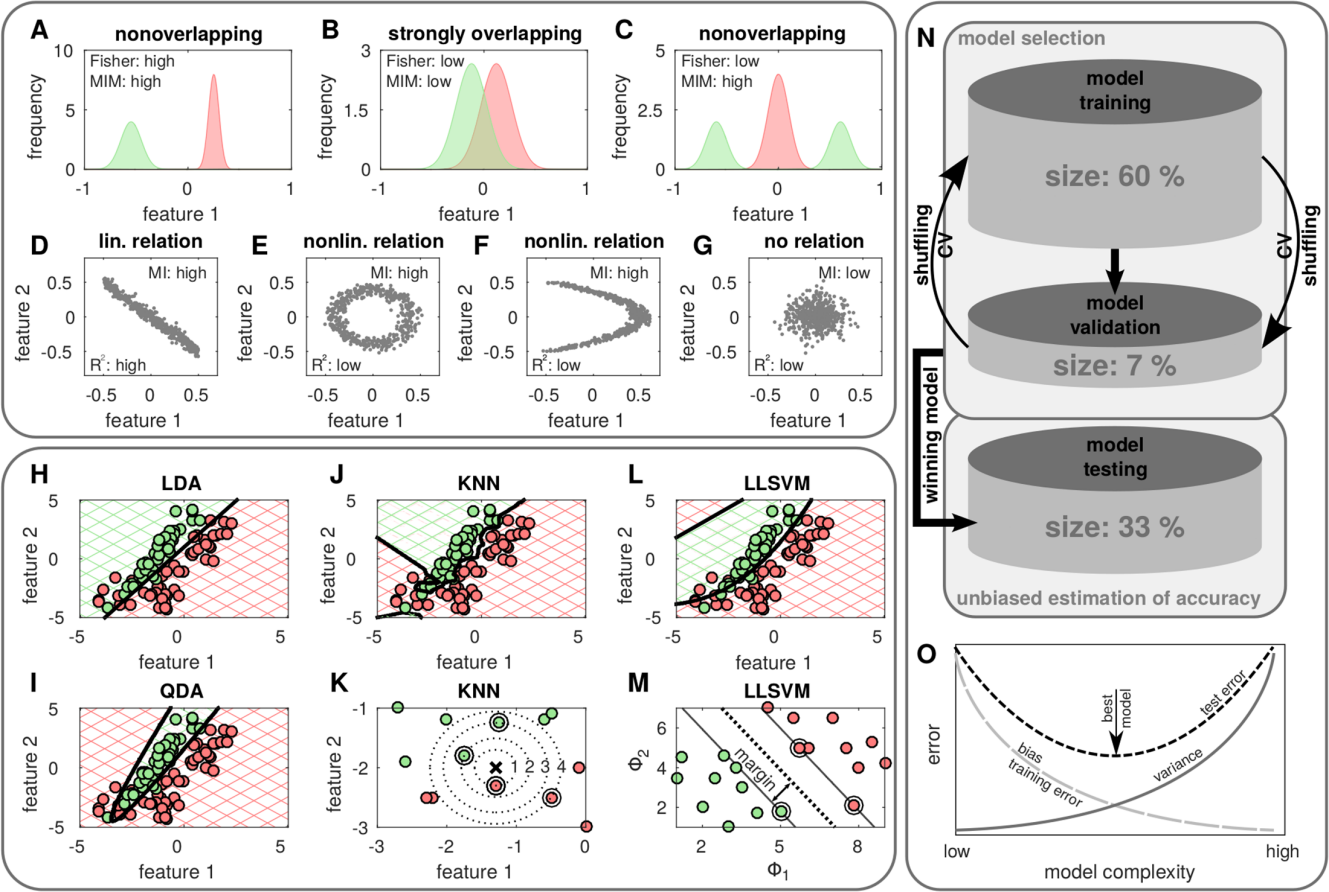
From feature to model selection. (A-C) Mutual information maximization (MIM) and Fisher criterion applied on linear separable and nonseparable two-class problems (red and green). (D-G) In contrast to the correlation coefficient mutual information (MI) recognizes linear and nonlinear dependencies between random variables. (H-M) The classification of a two class problem (red and green) using classifiers with different complexities display variations concerning the decision boundary. (N) The reshuffling of training and validation set (cross validation) helps to judge the model stability. For a performance check, the best model is used to classify the test set, which consist of so far unseen data not used for model selection. (O) To find the model best suited for discrimination (black arrow) the sum of two sources of errors (black, dashed) has to be minimized. Bias (light gray, dashed) decreases with increasing model complexity, whereas variance (dark gray, solid) increases.

Note, that this representation is valid for discretized features *χ*_*i*,*j*_ ∈ *χ*_*i*_ with *P* denoting its probability, *j* the discretization index and *k* its class belongings. In general, mutual information can be understood as the information gain of one random variable (*e.g.* class) if another one (*e.g.* features) is known. Hence, features with large values of *I* signal high discriminative power while low values mark noninformative features. For illustration, three simple one-dimensional examples showing the probability distribution of two classes are presented in Fig 2A–2C. MIM assigns high scores to the clearly separated distributions while strong overlapping distributions achieve a low score. In contrast to other correlation measures, such as the *R*^2^ value representing the squared correlation coefficient, mutual information is able to recognize nonlinear interrelations between random variables, see Fig 2D–2G.

### Maximum relevance minimum redundancy

The maximum relevance minimum redundancy (MRMR) [15, 16] is a multivariate information theoretic criterion taking dependencies between features into account. The selection process is a stepwise procedure adding one feature at a time to the set of selected features ***S***. Therefore, the first feature is selected by the MIM criterion. The next feature added to ***S*** is the one which maximizes the MRMR score Θ

$$\begin{array}{c} \Theta \left(\chi_{i},c\right)=I\left(\chi_{i},c\right)-\frac{1}{\left|S\right|}\sum_{s\in S}I\left(\chi_{s},\chi_{i}\right). \end{array}$$ (2)

After updating ***S*** the scheme is repeated until the number of desired features is reached. The first term in Θ measures the correlation between feature and class (relevance), whereas the second term penalizes correlations between the feature *χ*_*i*_ and the features belonging to ***S*** (redundancy).

### Fisher score

The Fisher score Ψ is based on the idea that features containing much discriminative power assign similar values to samples of one class while samples of other classes are assigned distinct values [17]. These features have class means being far apart compared to the standard deviation of each class. In mathematical terms this can be expressed as

$$\begin{array}{c} \Psi \left(\chi_{i},c\right)=\frac{\sum_{k}m_{k}{\left(\mu_{i,k}-\mu_{i}\right)}^{2}}{\sum_{k}m_{k}\sigma_{i,k}^{2}}, \end{array}$$ (3)

where *μ*_*i*_ and *σ*_*i*_ denote the mean and standard deviation of the *i*th feature with class belongings *k*. Hence, the statistical quantities *μ*_*i*,*k*_ and *σ*_*i*,*k*_ are computed using only *m*_*k*_ cells belonging to class *k*. The Fisher score, as a univariate approach, cannot capture feature dependencies. As a consequence, it assigns each feature a score relying on its intrinsic properties. In Fig 2A–2C the Fisher score is illustrated for three one-dimensional examples. It accurately identifies the high discriminative power of the nonoverlapping feature and the low discriminative power of the overlapping feature, but fails to correctly identify the discriminative power of the features of the linear nonseparable case.

## The problem of classification

Machine learning provides an efficient way for the automated classification of heterogeneous populations. Therefore, the class belongings of samples characterized by various properties are predicted. The prediction is an algorithmic procedure considering only the samples’ features. In the biological domain of cell state screening, classification is the prediction of cell states ***c*** based on features characterizing each single cell. Hence, it can be defined as a mapping *f* assigning samples from the *n*-dimensional feature space to a particular value of the categorical classes ***c***

$$\begin{array}{c} f\left(x\right):R^{n}\to \left\{c_{1},\ldots ,c_{n_{c}}\right\}. \end{array}$$ (4)

The most satisfying approach for classification would be to associate samples to the class, which is most probable regarding their particular feature realization ***x***. Unfortunately, it is in general not possible to reconstruct the underlying probability distribution, which is why assumptions regarding the distributions are made or heuristic classification criteria are applied.

### Discriminant analysis

Linear and quadratic discriminant analysis (LDA and QDA) assign class labels to unseen objects based on the class conditional probability distribution *P*(***x***|***c***). Applying Bayes’ rule the class is chosen, which maximizes the posterior probabilityc

$$\begin{array}{ccc} P(c|x) & = & \frac{P(x|c)P(c)}{P(x)}. \end{array}$$ (5)

If equal class priors are assumed, *e.g.* if no knowledge concerning the class frequency is available, this simplifies to the following optimization problem

$$\begin{array}{ccc} f(x) & = & \text{arg}\;\underset{c_{k}\in c}{\text{max}}\;P\left(x|c_{k}\right). \end{array}$$ (6)

The class conditional probability distribution of the feature vector ***x*** are assumed to be multivariate Gaussians with class dependent mean and (*i*) a single covariance matrix pooled for all classes leading to a linear separation boundary (LDA) or (*ii*) class dependent covariance matrices leading to a nonlinear separation boundary (QDA). Both methods are very robust concerning noise and outliners and easily applicable, since they do not depend on additional parameters [18]. In Fig 2H and 2I the classification results of both methods is shown for a linear nonseparable problem. In this case the QDA with its nonlinear separation boundary is able to discriminate the different classes more accurately than the LDA.

### *k*-nearest neighbors classifier

The *k*-nearest neighbors (KNN) classifier uses the class label information of the KNN of the training data to predict the label of unseen objects. Therefore, the majority class of the KNN is depicted for the assignment [19]. In most cases and also in this manuscript, the Euclidean metric is used to measure distances, but other measures can be applied as well [20]. In Fig 2J the results for the KNN classification are illustrated for the linear nonseparable example. The KNN classifier correctly recognizes the nonlinearity of the problem *via* a rough separation boundary. The more neighbors are used for the classification of an unknown object (black cross) the more distant points influence the prediction resulting in a smoother separation boundary, see Fig 2K. The KNN classifier is very simple and has the advantage that it does not depend on any assumptions of the underlying distribution. Its drawback is the huge computational load, especially for large training sets, since the distances to all samples have to be computed. Thus, the complexity of the algorithm scales with O(n×m) [21].

### Support vector machines

In order to discriminate samples belonging to different classes, the SVM maps the feature space *via* a nonlinear transformation Φ(***x***) to a higher-dimensional space leading to a model of the form

$$\begin{array}{c} f\left(x\right)=\text{sgn}(w^{⊤}\Phi \left(x\right)+b) \end{array}$$ (7)

with the weight vector ***w*** and the offset *b* [5, 22]. The nonlinear transformation can be efficiently computed by applying the “kernel trick” with *e.g.* Gaussian or polynomial kernels *κ* using the dual formulation [23]

$$f(x)=\sum_{i=1}^{m}y_{i}\alpha_{i}\kappa \left(x_{i},x\right)+b$$ (8)

$$\kappa (x_{i},x)=\Phi {\left(x_{i}\right)}^{⊤}\Phi \left(x\right).$$ (9)

***α*** denotes Lagrange multipliers and *i* indicates the *i*th sample. The classification of the two-class problem using a SVM with a Gaussian kernel is shown in Fig 2L. The separation boundary is set in a way that maximizes the margin between the two classes, which is defined as the smallest distance between the separation boundary and any of the samples from the training data, see Fig 2M. The samples located on the margin are called support vectors (circled dots). In the case of overlapping class conditional probability distributions, an exact separation might lead to a poor generalization. To forestall this incident and regularize the classifiers complexity, misclassification is allowed assuring a soft and smooth margin. Therefore, samples which are situated on the wrong side of the separation boundary or inside the margin are penalized. Since SVMs are very complex ($O(n\times m\times n_{c})$ [24]) and require huge memory for large scale data sets, we use an approximate version, which is called low-rank linearization support vector machine (LLSVM) [25]. For that reason the nonlinear kernel SVM is transformed into a linear one *via* decomposition of the kernel matrix *κ*(***x***_*i*_, ***x***_*j*_) using the Nyström method combined with *k*-means clustering [24, 25].

## Model selection

Models used for machine learning purposes generally depend on several hyperparameters. Since *a priori* the optimal values of these parameters are unknown, a set of competing models is on hand for classification. The task of model selection is to identify the model which discriminates the different classes in an optimal way meaning to predict the class labels of unknown data correctly. To estimate the model’s performance we introduce the 0-1 gain Γ

$$\begin{array}{c} \Gamma \left(y_{i},f\left(x_{i}\right)\right)=\{\begin{array}{cc} 1 & y_{i}=f\left(x_{i}\right)\\ 0 & \text{else} \end{array}, \end{array}$$ (10)

which assigns 1 if the sample ***x***_*i*_ is classified correctly and 0 if not. The average over all samples can be used to compute the accuracy Δ as a measure of performance

$$\begin{array}{c} \Delta =\frac{1}{m}\sum_{i=1}^{m}\Gamma \left(y_{i},f\left(x_{i}\right)\right). \end{array}$$ (11)

To find the best model, the data set ***D*** with known class labels is divided into two parts ***D***_*MS*_ ⊂ ***D*** used for model selection and ***D***_*test*_ = ***D***\***D***_*MS*_ to test the true prediction performance, see Fig 2N. We follow the strategy to train all competing models and roughly estimate their performance using ***D***_*MS*_. Applying a cross validation (CV) scheme to assess the model stability ***D***_*MS*_ is further divided into *K*_*CV*_ subsets $D_{MS,1},\ldots ,D_{MS,K_{CV}}$ of equal size and class composition (stratification). The model is trained *K*_*CV*_-times with *K*_*CV*_ − 1 subsets (training set) and each time validated on a different subset left out (validation set). By averaging over all CV iterations the model which achieves the highest accuracy will be regarded as optimal and used for further analysis and investigation. Since the accuracy is attained by using the data on which the model was build it might be biased and overoptimistic. Hence, an additional control on the independent test set ***D***_*test*_ is necessary to estimate the model performance correctly.

The performance of the classification techniques is heavily affected by features which differ in several orders of magnitude. Hence, transformation steps, such as scaling to a fixed mean and variance (standardization) or normalization to a particular interval, should be applied. Best practice is to scale the data used to train the classification scheme and apply the same transformation using the characteristics from the training set on the data which will be classified, see S1 Text.

The search for the optimal model constitutes an optimization problem in the space of hyperparameters [26]. Depending on the applied method these include the number of KNN, the regularization constant *C* and the kernel bandwidth *γ* of the SVM. Since the features are ranked by filtering, we can efficiently determine the best feature subset by treating the number of selected features *n*_*FS*_ as an additional hyperparameter. For the purpose of finding the global optimum, several strategies to explore the parameter space can be applied. Problems with only a few hyperparameters are often optimized by grid search [27], but also other techniques including genetic algorithms [28], particle swarm optimization [29] and random sampling approaches [27, 30] are suitable.

In order to select the model which captures the underlying relation between the classes best the sum of two sources of errors has to be minimized [31], see Fig 2O. The machine learning algorithm chosen for classification accounts for a systematic error, since it does not perfectly describe the underlying relation. This implies that bias regarding the algorithms designation to a particular set of functions is introduced. Additionally the classification is affected by the particular samples chosen for training, measurement noise and random behavior of the algorithm itself. The sensitivity to the initial realizations of these variables is called variance. The tradeoff between bias and variance is necessary to select the optimal model minimizing the validation error, see Fig 2O. Models which are too complex suffer from overfitting modeling random fluctuations, whereas the models which are too simple miss underlying relations (underfitting).

## Results

In the following, we investigate two independent IFC experiments with the aim to distinguish viable and apoptotic cells. Apoptosis can be induced by intrinsic or extrinsic stimuli [32] and is characterized by the activation of caspases, cell shrinkage, membrane blebbing and nuclear fragmentation [33]. Hence, apoptotic cell death is commonly detected using various fluorescence dyes that specifically target morphological changes of dying cells, such as loss of membrane integrity [34]. Recent approaches indicate that morphological features provide useful information for the classification of apoptosis using IFC data [35]. In case study 1 we apply our machine learning approach on morphological features extracted from bright and dark field images to discriminate between dying and living cells. Thereby, we uncover a number of advantages compared to the classical apoptosis detection approaches of Annexin V and propidium iodide (PI) staining. Afterwards, we demonstrate in case study 2 using intracellular staining of fixed cells which pitfalls are frequently encountered in automated classification.

### Case study 1

The traditional two-dimensional gating strategy utilizing the dyes Annexin V and PI is very accurate and well established in the community [34]. However, automated machine learning approaches purely based on morphological features represent a charming alternative. The morphological features are extracted from bright and dark field images and characterize cellular attributes, such as size, shape and texture. Many of these features correlate to morphological changes which are also targeted by cell death specific staining. By demonstrating the conformity of both methods we show that Annexin V/PI staining can be replaced by noninvasive machine learning techniques on label-free data.

**Fig 3:**
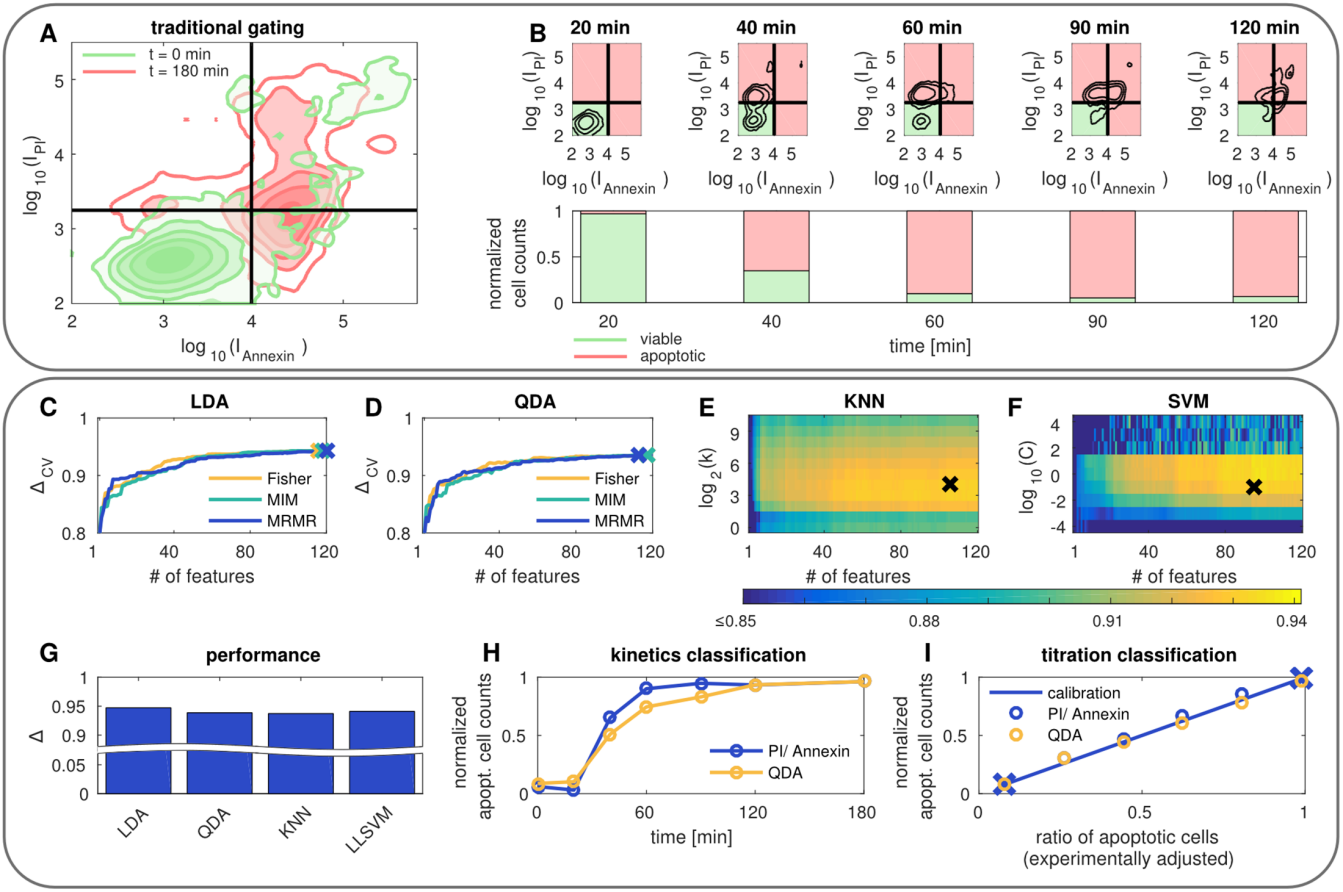
Traditional gating on labeled data *vs.* machine learning on morphological properties. (A) Two-dimensional gating approach for Annexin V and propidium iodide (PI) staining. (B) Classification of kinetics data using the gates determined by A. (C-F) CV accuracy as function of hyperparameters for different feature selection and classification schemes. The crosses indicate the winning models. (G) Unbiased model performance of the winning models. (H-I) Comparison of the traditional gating technique (blue) and the machine learning approach (yellow) for kinetics data and titration data. The crosses in I mark the ratios obtained from A, which are used for calibration.

The data used in this case study consists of two different kinds of measurements. The first measurement comprises nonstimulated cells which are nearly all viable, whereas the second measurement comprises CD95-stimulated cells. Stimulation of the death receptor CD95 triggers caspase activation and apoptosis induction. In our study, we used a high dosage of CD95L resulting in the large fraction of apoptotic cells after 180 min of stimulation. Applying the traditional gating approach using cell death specific dyes yields the classification scheme illustrated in Fig 3A. The trained model can then be applied to data with unknown class belongings to discriminate viable and apoptotic cells, see Fig 3B illustrating the temporal evolution of a dying cell population.

To train the machine learning approach, we use the class belongings (viable or apoptotic) obtained by the traditional method and the so far unused morphological features. The cell death specific dyes Annexin V and PI are not used. For visual purposes we explored the hyperparameter space and selected the best model by grid search. LDA and QDA are very simple classifiers meaning that the only parameter which has to be optimized is the number of features, see Fig 3C and 3D. To fine tune the more complex classifiers, additional parameters have to be adjusted. These comprise for the KNN classifier the number of neighbors *k* and for the LLSVM using a Gaussian kernel the regularization parameter *C* as well as the kernel band width *γ*, see Fig 3E and 3F and S1 Text. Interestingly, there was no significant difference in the performance of the feature selection techniques being used, see S1 Text. The unbiased accuracy estimated from the test set is presented for the winning models in Fig 3G. All of them achieve accuracies of approximately 95%, which comes very close to the traditional approach. Similar to the two-dimensional gating technique on labeled data the machine learning models based on morphological features can be applied on cells with unknown class belongings. In Fig 3H the results of the QDA on kinetics data are shown vicarious for all classification schemes. It turns out that the automated machine learning approach yields comparable results to the traditional gating method. Also regarding titration measurements with experimentally adjusted ratios of viable and apoptotic cells similar results were obtained, see Fig 3I and S1 Text. The QDA and the traditional approach are very close to the calibration curve obtained by using the data shown in Fig 3A. Hence, machine learning on label-free data is an excellent alternative to cell death specific staining regarding the characterization of the dynamic process of apoptosis.

### Case study 2

In the second case study, we regard an experiment where three different dyes are measured additional to the morphological features extracted from the bright and dark field images. The cells are stimulated with CD95L and stained with Zombie Aqua, which indicates loss of membrane integrity. After fixation and permeabilization, the cells were stained with an antibody against cleaved caspase-3 and the DNA dye 7AAD. We use this data set to illustrate which difficulties might occur when using automated machine learning classification and how to avoid several pitfalls that frequently arise. Our investigation focuses on three particular examples of the following issues: (*i*) the impact of imbalanced data on performance estimation, (*ii*) the identification of low quality features obtained from fluorescence dyes and (*iii*) how usage of biological knowledge supports model selection.

#### Imbalanced data

**Fig 4:**
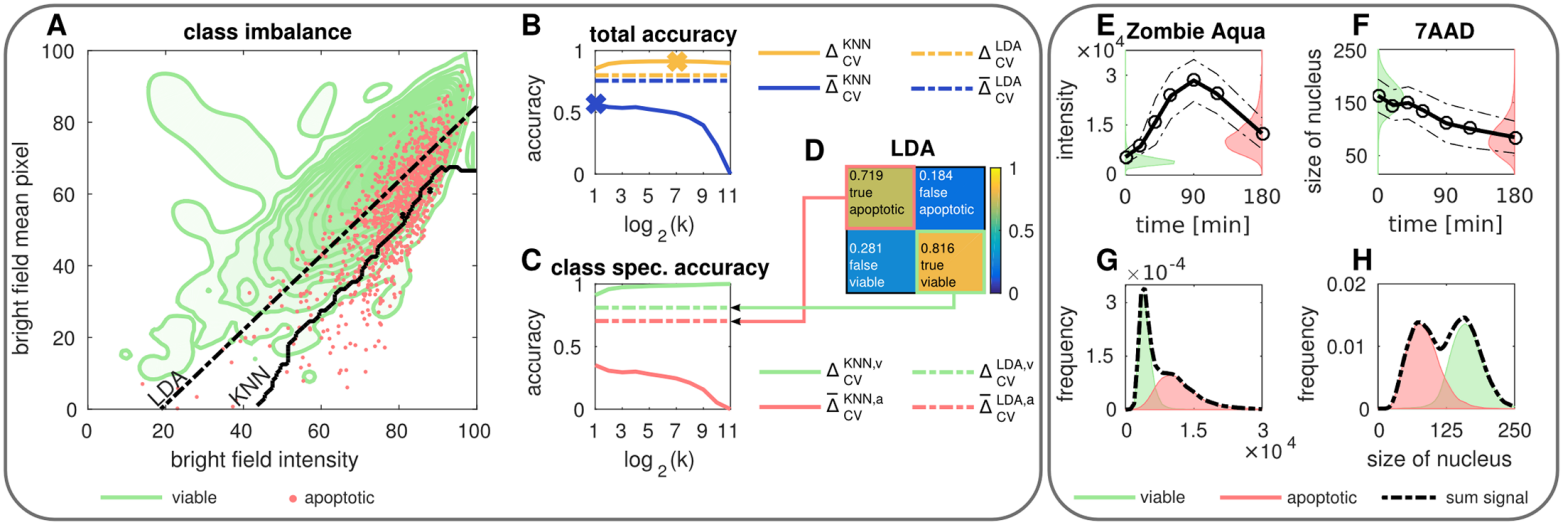
Impact of class imbalance and feature quality on classification. (A) Two-dimensional representation of viable (green) and apoptotic (red) cells with corresponding separation boundaries obtained by LDA (black, dashed) and KNN classification (black, solid). (B) Comparison of CV accuracy (yellow) and its class specific geometric mean (blue) of LDA (dashed) and KNN classification (solid) as measure of performance. (C) Class specific CV accuracy of viable (*v*, green) and apoptotic (*a*, red) cells for LDA (dashed) and KNN classification (solid). (D) Class specific confusion matrix obtained by LDA. (E-F) Temporal evolution of the size of the nucleus measured by 7AAD and loss of membrane integrity measured by Zombie Aqua. (G-H) Comparison of viable (green) and apoptotic (red) distribution of the size of the nucleus and loss of membrane integrity.

To build up the model two measurements were used: (*i*) nonstimulated cells and (*ii*) cells that have been stimulated for 180 min with CD95L. Since we did not measure cell death with the traditional gating method described in the previous section, we assume that in the measurement of unstimulated cells all cells are viable and in the measurement of stimulated cells all cells are apoptotic. From the previous case study we know that this is a valid assumption, see Fig 3H. The ratio of apoptotic to viable cells is approximately $\frac{1}{10}$. Hence, the data set comprises class imbalance (skewness). Models based on such imbalanced data often favor the majority class [36, 37] as seen in Fig 4A. In this case a two-dimensional example with strongly overlapping populations is illustrated. The contour plot characterizes the viable class, whereas the sparsely sampled apoptotic class is presented by a dot cloud. We used an LDA and a KNN classifier to discriminate the classes. For the KNN classifier *k* is determined by optimizing the CV accuracy analogue to the previous section, see Fig 4B. It turns out that compared to the LDA the nonlinear classification *via* the KNN classifier achieves a higher CV accuracy, which is not very sensitive to the particular value of *k*. The LDA, however, does not depend on any hyperparameter and is therefore illustrated as a constant line. When examining the separation boundaries in detail we notice that although the LDA achieves a lower accuracy it recognizes the underlying distribution better. The KNN classifier assigns nearly all samples to the majority class, since the training data is heavily skewed. This can be seen in Fig 4C illustrating the class specific CV accuracy for the LDA and the KNN classifier. Here we observe for the KNN classifier a strong increase in correctly predicted class belongings of viable cells for increasing *k*. In contrast we register a decline of correct class predictions of apoptotic cells. For the LDA only a small gap between the correct predictions for both classes is visible meaning that viable and apoptotic cells are recognized approximately equally well. The corresponding confusion matrix is illustrated in Fig 4D presenting correct classification and misclassification.

The geometric mean $\bar{\Delta }=\sqrt{\Delta_{v}\Delta_{a}}$ of the class specific accuracies represents an alternative metric for performance estimation. The subscripts *v* and *a* denote in this case the viable and apoptotic classes. In contrast to the accuracy Δ, the geometric mean $\bar{\Delta }$ reflects the low performance of the KNN classifier for high values of *k*, see Fig 4B and 4C. The LDA is not very sensitive to class imbalance, since only mean and covariance are estimated from the distributions. Therefore, accuracy and geometric mean are very close to each other and yield similar results. It turns out that the CV accuracy as a measure of performance is only adequate if the abundance of both classes is balanced. For skewed problems, performance should be measured using a metric which adequately weights and balances the class specific accuracies. Otherwise the minority class might not be discriminated accurately.

#### Feature quality

Next to Annexin V/PI staining other strategies for apoptosis detection, such as the monitoring of loss of membrane integrity [38] and size of the nucleus [35], have been proposed. To verify their eligibility regarding the classification approach we investigate their temporal correlation to apoptosis. The viability dye Zombie-Aqua can enter cells only if the membrane is demolished, *e.g.* due to cell death. When studying the temporal dynamics of a dying cell population the Zombie-Aqua intensity increases with time as expected, but after a certain time point it drops, see Fig 4E. In this case the cell destruction most probably resulted in the outflow of the dye or cellular protein content causing the decrease of intensity. The comparison of the intensity distribution of the viable and apoptotic cell population reveals a strong overlap, see Fig 4G. In an experiment Zombie-Aqua is used to discriminate viable and apoptotic cells in a population of unknown composition, see dashed distribution in Fig 4G. This unimodal distribution can be hardly dismantled into its components (viable and apoptotic), and therefore does not contain much information on its own. In contrast, the DNA dye 7AAD is highly useful to monitor the size of the nucleus by measuring the area of the 7AAD signal, see Fig 4F. It is well known that the process of apoptosis goes along with nuclear fragmentation and shrinkage [33, 39], which can be clearly observed by means of a declining trend regarding the size of the nucleus, see Fig 4F. When conferring the nuclear size of viable and apoptotic cells, we recognize a small overlap between nonstimulated and apoptotic cells, see Fig 4H. Despite this, the sum distribution results in a bimodality supporting the discrimination of viable and apoptotic cells.

Fluorescence dyes are usually used to enhance the interpretability of biological experiments. Although many staining products for color based classification are published to work in some cases, they might fail in a particular application. In order to prevent such failures, we recomment to check the suitability of features obtained *via* staining. In this case we identify the low discriminative power and implausible temporal evolution of Zombie-Aqua. To avoid disturbance of the classification scheme by the artificial Zombie-Aqua signal we recommend to exclude it for further analysis.

#### Biological knowledge

**Fig 5:**
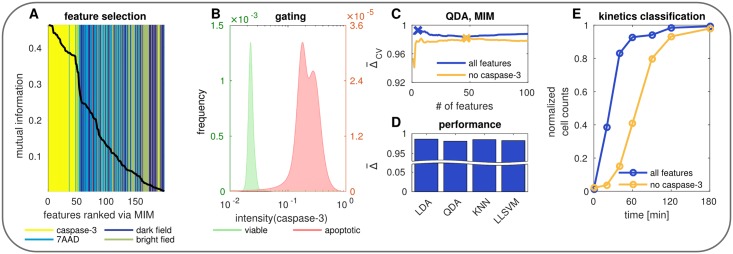
Information content of caspace-3. (A) Feature selection *via* MIM. (B) Normalized caspase-3 intensity of viable cells without stimulation (green) and apoptotic (red) cells 180 min after stimulation. (C) Model selection for all features (blue) *vs.* all features except caspase-3 (yellow). (D) Performance comparison of different classification algorithms trained on all features except caspase-3. (E) Classification of kinetics data using the optimal models from C.

In order to complement black-box machine learning approaches and facilitate the identification of promising and unsuitable features, biological knowledge should be used (if available) [40]. In our case study of apoptosis we are aware that active caspase-3 is the executioner of apoptosis [32]. A rise in the amounts of active caspase-3 will trigger the cleavage of essential downstream proteins leading to apoptosis [32]. As expected, feature selection showed a strong correlation of caspase-3 activation and apoptosis. In Fig 5A the features are ordered by MIM with color coded measurement dependency. We observe that approximately the forty most important features all belong to caspase-3 indicating its immense information content. For the remaining features such behavior is not visible meaning that the features of 7AAD, bright and dark fields are well mixed regarding their ranking *via* MIM. To emphasize the huge information content of caspase-3 we illustrated the distribution of its intensity in Fig 5B showing two nearly perfectly separated peaks.

In order to investigate the impact of caspase-3 on the classification performance, we first compare two classification schemes *i.e.* using all features and using all features except caspase-3 for model building, see Fig 5C. We depict this using a QDA for classification and MIM for features selection. It turns out that the scheme using all features slightly outperforms the one omitting caspase-3, but both yield excellent results. The best models are marked by a cross. When we compare the unbiased performance of the QDA omitting caspase-3 to other classification algorithms, no significant difference can be observed, see Fig 5D.

To verify the suitability of the caspase-3 features we compare both models on kinetics data resulting in large deviations, see Fig 5E. The ratio of apoptotic cells using caspase-3 features resembles a hyperbolic function, whereas the ratio of apoptotic cells not using caspase-3 features depicts a sigmoid curve, as expected. Especially for the early time points (20, 40, 60 min) large differences are visible. Nonstimulated (0 min) and nearly completely late apoptotic (180 min CD95L) cells used for performance estimation showed a good agreement of apoptosis estimation for both methods. This demonstrates that caspase-3 features are suitable to discriminate stationary populations, but fail to accurately characterize the dynamic process of apoptosis. Hence, caspase-3 features should be taken with care when it comes to classification of dynamic data.

It is well known that morphological changes in the course of apoptosis occur later than caspase-3 activation [35], since there is a causal relation between both effects [32]. First caspase-3 is activated and subsequently downstream processes are triggered, which lead to apoptosis. As a consequence, with the aid of biological knowledge caspase-3 could be identified right from the beginning as a feature with high potential for misinterpretation. By pure computational analysis this drawback of caspase-3 staining would not be recognized.

## Discussion

Starting with the classification problem of high-dimensional biological IFC data, we elucidated popular machine learning approaches and analyzed them in two case studies of apoptosis detection. We compared our results using label-free classification to traditional Annexin V/PI staining and demonstrated conformity of both methods regarding the characterization of the dynamic process of apoptosis. The automatization of complex tasks such as discrimination of cellular phenotypes is not straightforward and potential perils are often overlooked. To address this issue we high-lighted the necessity to include prior knowledge for identification of misleading features measured by fluorescence dyes. Thereby, we uncovered undesirable behavior of Zombie Aqua and caspase-3 dye for apoptosis detection regarding the analysis of kinetics data. By exclusive reliance on model selection these effects would not have been recognized. Furthermore, we illustrated how to cope with class imbalance and revealed its impact on model performance. Although the classification accuracy is often used as metric for performance estimation it can fail for highly skewed problems. Hence, we recommend to apply the geometric mean of the class specific accuracy for data comprising class imbalance.

Through this study different techniques for feature selection and classification were used, which vary significantly in computational effort. The comparison of uni- and multivariate feature selection algorithms did not show significant differences regarding the performance. Both techniques performed equally well, although often differences in the number of selected features occured, see S1 Text. Regarding the classification schemes utilized in this study we noticed that the model selection of LDA and QDA was much faster compared to the highly nonlinear classifiers, since the number of features was the only hyperparameter being optimized. Often the highly nonlinear classifiers choose fewer amounts of features, however, all classification schemes yield similar performances. Condensing the outcome on the two case studies presented here we emphasize that often, especially in the context of big data, simple linear methods yield excellent results while being computationally very efficient. We therefore advise to apply the univariate feature selection with LDA and QDA before thinking of very complex classification schemes, in other words: Keep It Simple.

The ability of single cell measurement techniques such as IFC to collect vast amounts of data urged for novel approaches regarding modeling and data analysis. Especially the label-free cell state screening of big data IFC measurements has become an important topic in computational biology and bioinformatics. Hence, several approaches for the analysis of IFC data relying on different techniques for dimensionality reduction, model selection and classification have been proposed recently [6, 7, 41, 42]. However, our approach allows to efficiently incorporate feature selection in the process of model selection by treating the number of selected features as a single hyperparameter, which has to be optimized. Thus, our approach stands out due to its simplicity and applicability. In order to give additional insight we provide MATLAB code for the illustration of our findings [43]. By editing the code our universally applicable approach can be transfered to different problems not restricted to apoptosis detection in HeLa cells, *e.g.* stem cell proliferation or cell cycle analysis. The choise of the cell line and the process being monitored do not alter the applicability of our approach as long as it can be characterized well by morphological changes. Since our approach is not limited to imaging flow cytometry data other measurement devices, such as confocal microscopy, can be used. Therewith it is possible to monitor three-dimensional morphological changes by combining features of two-dimensional images taken at different focus depths.

## Materials and methods

### Experiments

#### Cell culture

HeLa-CD95 cells were described in [44] and cultivated in DMEM/HamsF12 media (Biochrom, Berlin, Germany) supplemented with 10% FCS (Lifetechnologies, Darmstadt, Germany), 1% Penicillin/Streptomycin (MerckMillipore, Darmstadt, Germany) and 10 ng/ml Puromycin (Sigma Aldrich, Taufkirchen, Germany). Cells were grown at 37°C and 5 CO_2_.

#### Parallel measurement of cell death and caspase-3 activation

On the day before stimulation 5 × 10^5^ HeLa-CD95 were plated in 6 cm dishes. The next day, the media was replaced with 1 ml of fresh media and cells were stimulated with 250 ng/ml CD95L. After the stimulation media was removed, cells were washed with PBS and detached by trypsination for 5 minutes at 37°C. Media, PBS and cells were collected and spun down at 500 × *g* and 4°C for 5 minutes. Supernatant was removed and cells were suspended in 1 ml PBS. One half of the cells were fixated for staining with antibodies while the other half was used for measuring cell death with annexin V/ PI staining.

For fixation, the cells were spun down as before and suspended in 100 *μl* PBS. 0.5 *μl* Zombie Aqua dye (BioLegend, city, USA) were added for 20 minutes at room temperature (RT) in the dark. After this, the cells were spun down as before and suspended in 250 *μl* PBS. Next, 250 *μl* 6% formaldehyde in PBS was added slowly and the cells were incubated for 10 minutes at RT in the dark. The cells were spun down at 500 × *g* and 4°C for 5 minutes and the pellet was suspended in −20°C cold 90% methanol, incubated on ice for 30 minutes and stored at −20°C over night. The next day, the cells were spun down at 500 × *g* and 4°C for 5 minutes and washed twice with 0.5 ml incubation buffer (5g/l BSA in PBS). After washing, the pellet was supended in 50 *μl* incubation buffer. After 10 minutes at RT in the dark anti-cleaved-caspase-3 alexafluor647 antibody (CellSignalingTechnologies, city, USA) was added. After 1 hour incubation at RT in the dark, the cells were washed with 0.5 ml incubation buffer again and suspended in 40 *μl* PBS. At least 5 minutes before measuring 3 *μl* 7AAD (BioLegend, city, USA) were added. The cells were measured with Amnis FlowSight (MerckMillipore, Darmstadt, Germany). 7AAD was measured in channel 5 (642-745 nm), Zombie Aqua in channel 8 (505-560 nm) and alexafluor647 in channel 11 (642-745 nm).

Non fixated cells were spun down at 500 × *g* and 4°C for 5 minutes and were stained with 3 *μl* AnnexinV-FITC (Immunotools, city, country) and 2 *μl* PI in 100 *μl* annexin staining buffer (Roche, city, country) for 15 minutes in the dark. After spinning down as before the cells were suspended in 50 *μl* of annexin staining buffer and measured with Amnis FlowSight. FITC was detected in channel 2 (505-560 nm), PI was detected in channel 4 (595-642 nm) and bright field acquired in channel 1 (435-505 nm). For all experiments automated compensation was done with single stained samples. Only focused images of single cells were selected for further processing.

### Data analysis

The preprocessing and feature extraction was performed with IDEAS ImageStream Analysis Software. A list of features used for computation is provided in the S1 Text. The data analysis was performed using MATLAB 2015b. The code illustrating our analysis in case study 1 and 2 can be found in [43]. Since model selection *via* grid search is a tedious process for KNN and SVM due to their high complexity we provide code for efficient model selection using a genetic algorithm. We used the LLSVM implementation from [24].

## Supporting information

S1 Text Supplementary material containing detailed discussions and further information regarding our approach. (PDF)
