# Supplementary material for: A guide to automated apoptosis detection: How to make sense of imaging flow cytometry data
Source: PLoS One. 2018 May 16;13(5):e0197208. doi: 10.1371/journal.pone.0197208 (PMC5955558; doi:10.1371/journal.pone.0197208)
Supplement: S1 Text — (PDF) [file pone.0197208.s001.pdf]

SUPPORTING INFORMATION
A Guide to Automated Apoptosis Detection: How to Make Sense of Imaging Flow Cytometry Data

Dennis Pischel[1], Jörn H. Buchbinder[2], Kai Sundmacher[1, 3], Inna N. Lavrik[2], and Robert J. Flassig[3, *]

[1]Process Systems Engineering, Otto-von-Guericke-University Magdeburg, Magdeburg, Germany
[2]Translational Inflammation Research, Otto-von-Guericke-University Magdeburg, Magdeburg, Germany
[3]Process Systems Engineering, Max Planck Institute for Dynamics of Complex Technical Systems, Magdeburg, Germany
[*]Corresponding author contact: flassig@mpi-magdeburg.mpg.de

April 18, 2018

Contents

1 Workflow 1

2 Case Study 1 2

3 Model Selection *via* Genetic Algorithm 3

4 Case Study 2 3

5 MATLAB Implementation 3

1 Workflow

In order to give further insight regarding the proposed workflow a stepwise description is given here. We start with the partition of the whole data set $\boldsymbol{D}$ in a set used for model selection $\boldsymbol{D}_{MS}$ and a set used for testing $\boldsymbol{D}_{test}$. In the next step $\boldsymbol{D}_{MS}$ is divided *via* CV into K_{CV} parts. K_{CV} is set to 10, since a CV over all combinations is computational too intense and does not significantly improve the precision of the estimated accuracy Kohavi (1995). In each iteration of the CV the features of the training set are scaled

by subtracting the mean and dividing by the standard deviation. Therefore all features in the training set have zero mean and a standard deviation of one. The validation set is scaled by the same procedure using mean and standard deviation of the training set. Note, that these transformations do not belong to data preprocessing, but are an integral part of the model building process. The transformation of the whole data set during model selection is not recommended, since this introduces bias by using information from the test set for model selection. In principle the same proceeding pertains for feature selection. Therefore feature selection should be applied during CV resulting in the selection of different feature subsets in every iteration. In order to elude this difficulty we relax this proceedings and perform feature selection on $\boldsymbol{D}_{MS}$. Thus, bias might be introduced in model selection, but the accuracy estimated from the independent test set is still unbiased and trustworthy.

The classifiers are trained *via* grid search using all combinations of hyperparameters (*e.g.* number of features (all classifiers), number of nearest neighbors (KNN), regularization parameter (LLSVM), kernel band width (LLSVM)) and validated on the validation set for all feature selection schemes. We sampled the number of features equidistantly and the remaining hyperparameters logarithmically. By this we explored the performance in regard of the parameter space and are able to identify the best models achieving the highest performance. Especially for the highly nonlinear classifiers *i.e.* KNN and SVM, the exploration of the parameter space can be very time consuming, since the parameter space and training time of these classifiers is bigger compared to LDA and QDA. Note, that the grid like exploration of the parameter space can be replaced by other optimization schemes. After identifying the best hyperparameters the process of model selection is complete. To estimate the models' unbiased performance they have to be validated on the independent test set. Therefore $\boldsymbol{D}_{MS}$ and $\boldsymbol{D}_{test}$ are scaled as done before. The models using the best parameters from the model selection part are trained on $\boldsymbol{D}_{MS}$ and validated on $\boldsymbol{D}_{test}$ yielding the unbiased performance. To undertake further predictions (*e.g.* the classification of kinetics or titration data) we used the best models from the model selection part and retrained them on $\boldsymbol{D}$. By this we end up with the optimal model trained on the whole data set with an unbiased estimate of its performance.

2 Case Study 1

In the first case study 120 features extracted from bright and dark field images characterizing morphological properties of CD95 stimulated HeLa-CD95 cells were used. The cells were stained with Annexin V and PI without prior fixation, which does not allow the usage of antibodies for intracellular staining. A detailed description of the measured features can be found in Tab. 1. In the manuscript the CV accuracy of the KNN classifier and the LLSVM was only shown for certain values of hyperparameters and feature selection schemes. Therefore the whole analysis is presented in Fig. 1-2. The unbiased performance measured on the independent test set for all winning models is presented in Tab. 2. Their predictions on the kinetics and titration data is shown in Fig. 3.

3 Model Selection *via* Genetic Algorithm

As stated in the manuscript model selection can be understood as an optimization problem with the objective to maximize the classification accuracy. If the model depends only on a few hyper parameters as in our case, conventional grid search is a popular choice for optimization. To accelerate the process of model selection heuristic optimization schemes are often applied. In this section we demonstrate the effectiveness of a genetic algorithm. The genetic algorithm is a simple evolutionary optimization technique, which is inspired by natural selection. In Fig. 4 the model selection *via* a genetic algorithm with 20 generations and 8 individuals in each population is displayed. After a few generations the genetic algorithm finds the region of interest in the parameter space and converges to solution very close to the optimum computed with grid search. We provide a MATLAB script illustrating this optimization approach for KNN and LLSVM Pischel et al. (2018).

4 Case Study 2

In the second case study the feature ranking with color coded measurement dependency (bright field, dark field, 7AAD and caspase-3) was only shown for MIM. The ranking for all feature selection techniques is illustrated in Fig. 5. The performance for all classifiers on the test set excluding caspase-3 was only shown for the best models in the manuscript. In Tab. 3 the performance is presented for all feature selection techniques and classifiers. Their predictions on the kinetics data is shown in Fig. 6.

5 MATLAB Implementation

All results shown is this study were performed using MATLAB 2015b. In order to demonstrate our methodology we provide three scripts Pischel et al. (2018). Two scripts illustrate the classification strategy including feature selection, model selection, performance estimation and model application of case study 1 and 2. The third script shows the usage of a genetic algorithm for efficient model selection (case study 1).

Supplementary Tables

Table 1: List of features used in case study 1 and 2.

#	feature	descriptopn	CS
1	area	area [μm^2]	1,2
2	aspect ratio intensity	minor axis intensity divided by major axis intensity	1,2
3	modulation	intensity range	1,2
4	gradient rms	average gradient of a pixel	1,2
5	mean pixel	intensity divided by number of pixels	1,2
6	max pixel	largest value of pixels	1,2
7	raw min pixel	smallest value of pixels (no background subtraction)	1,2
8	width	width	1,2
9	bright detail intensity r3	intensity of localized bright spots	1,2
10	perimeter	boundary length of mask [μm]	1,2
11	compactness	compactness	1,2
12	lobe count	number of lobes	1,2
13	symmetry 2	tendency of lobe symmetry	1,2
14	symmetry 4	tendency of lobe symmetry	1,2
15	major axis	longest dimension of surrounding ellipsis	1,2
16	minor axis	shortest dimension of surrounding ellipsis	1,2
17	spot area min	area of smallest spot	1,2
18	thickness min	smallest width	1,2
19	angly intensity	angle of major axis intensity from horizontal plane	1
20	centroid x intensity	intensity weighted x centroid	1
21	centroid y intensity	intensity weighted y centroid	1
21	valley x	x coordinates of minimum intensity within skeletal mask	1
23	bright detail intensity r7	intensity of localized bright spots	1,2
24	h contrast mean	Haralick et al. (1973)	1,2
25	h correlation mean	Haralick et al. (1973)	1,2
26	h energy mean	Haralick et al. (1973)	1,2
27	h entropy mean x	Haralick et al. (1973)	1,2
28	h homogeneity mean	Haralick et al. (1973)	1,2
29	h variance mean	Haralick et al. (1973)	1,2
30	spot count	number of spots	1
31	area threshold	area of the nucleus	2
32	aspect ratio	minor axis divided by major axis	1,2
33	bkgd mean	average background	1
34	contrast	sharpness quality	1,2
35	intensity mc	intensity	1,2
36	median pixel	median of pixels	1,2
37	raw max pixel	smallest value of pixels (no background subtraction)	1,2
38	length	length	1,2
39	height	heigths	1,2
40	diameter	diameter	1,2
41	circularity	degree of deviation from a circle	1,2
42	elongatedness	heigth divided by width	1,2
43	shape ratio	thickness min divided by length	1,2
44	symmetry 3	tendency of lobe symmetry	1,2
45	min pixel	smallest value of pixel	1,2
46	major axis intensity	intensity weighted longest dimension of surrounding ellipsis	1,2
47	minor axis intensity	intensity weighted shortest dimension of surrounding ellipsis	1,2
48	thickness max	largest width	1,2
49	angle	angle of major axis from horizontal plane	1
50	centroid x	x centroid	1
51	centroid y	y centroid	1
52	spot distance min	shortest distance between two spots [μm]	1
53	valley y	y coordinates of minimum intensity within skeletal mask	1
54	gradient max	largest gradient of a pixel	1,2
55	h contrast std	Haralick et al. (1973)	1,2
56	h correlation std	Haralick et al. (1973)	1,2
57	h energy std	Haralick et al. (1973)	1,2
58	h entropy std	Haralick et al. (1973)	1,2
59	h homogeneity std	Haralick et al. (1973)	1,2
60	h variance std	Haralick et al. (1973)	1,2
61	std	standard deviation of pixels	1,2

Table 2: Model performance of case study 1.

classifier	Fisher Δ	Fisher hyperparameters		MIM Δ	MIM hyperparameters		MRMR Δ	MRMR hyperparameters	
LDA	0.947	n :	116	0.948	n :	118	0.947	n :	120
QDA	0.938	n :	117	0.939	n :	117	0.937	n :	113
KNN	0.933	n :	83	0.935	n :	93	0.937	n :	106
		k :	8		k :	8		k :	16
SVM	0.941	n :	95	0.948	n :	99	0.947	n :	102
		C :	10		C :	10		C :	1
		γ :	10^{-2}		γ :	10^{-2}		γ :	10^{-2}

Table 3: Model performance of case study 2.

classifier	Fisher Δ	Fisher hyperparameters		MIM Δ	MIM hyperparameters		MRMR Δ	MRMR hyperparameters	
LDA	0.986	n :	40	0.986	n :	44	0.985	n :	71
QDA	0.981	n :	42	0.979	n :	47	0.981	n :	93
KNN	0.984	n :	26	0.985	n :	47	0.984	n :	29
		k :	16		k :	8		k :	16
SVM	0.983	n :	39	0.980	n :	48	0.979	n :	55
		C :	10		C :	10		C :	1
		γ :	10^{-2}		γ :	10^{-2}		γ :	10^{-3}

Supplementary Figures

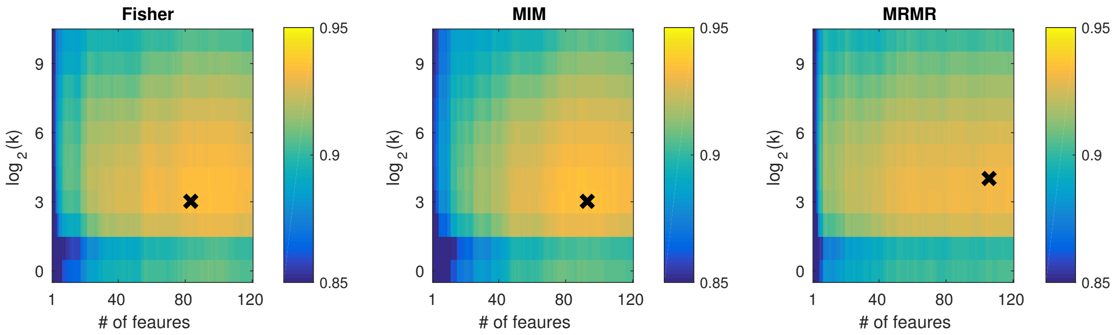

Figure 1: Model selection for KNN.

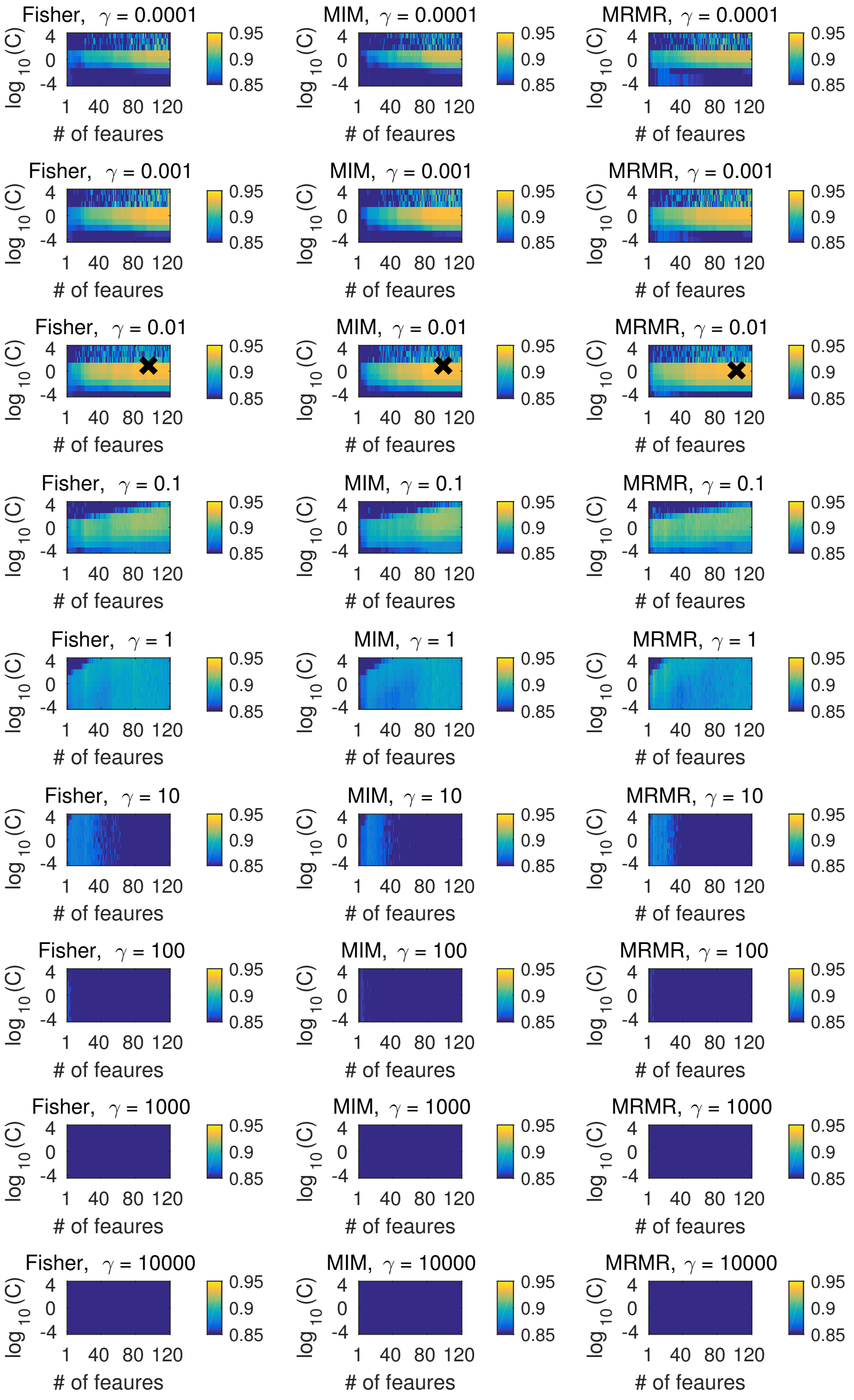

Figure 2: Model selection for SVM.

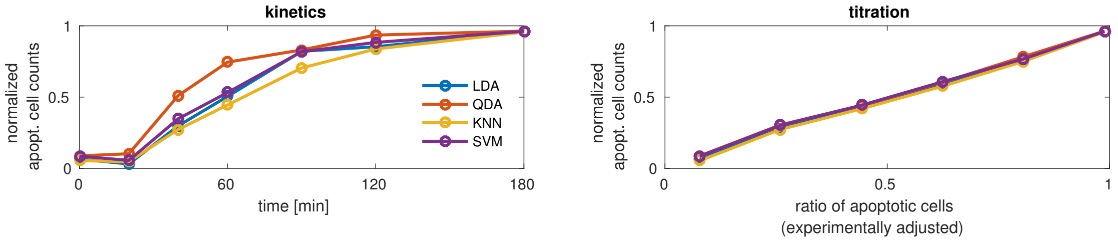

Figure 3: Model predictions on kinetics and titration data.

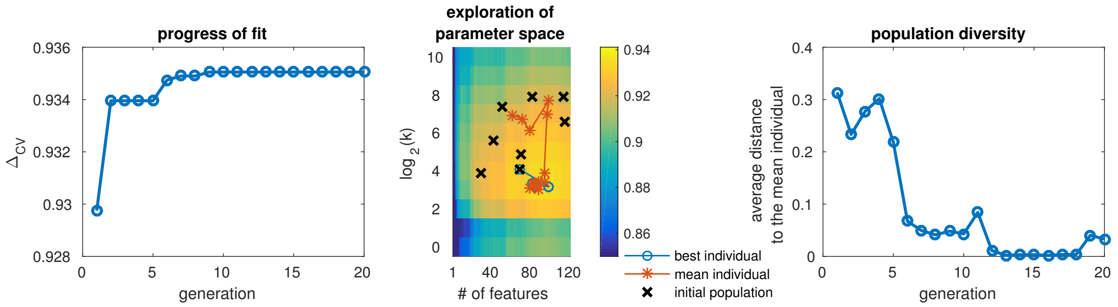

Figure 4: Model selection *via* genetic algorithm.

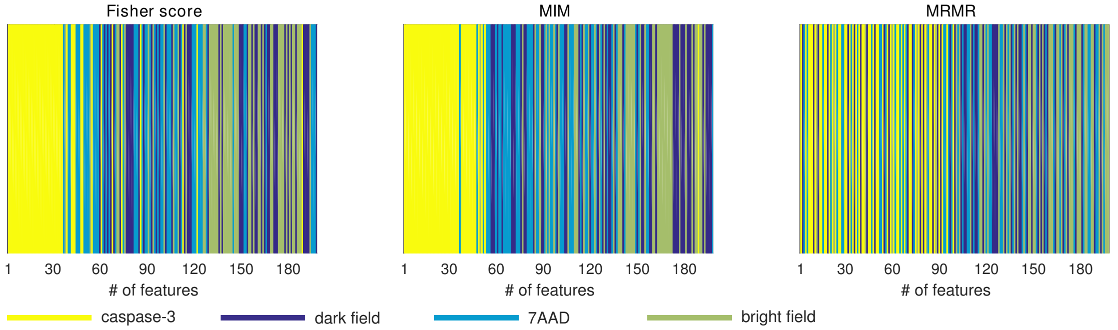

Figure 5: Feature ranking *via* Fisher score, MIM and MRMR.

References

R.M. Haralick, I. Dinstein, and K. Shanmugam. Textural features for image classification. *IEEE Transactions on Systems, Man and Cybernetics*, SMC-3(6):610–621, 1973.

Ron Kohavi. A study of cross-validation and bootstrap for accuracy estimation and model selection. In *Ijcai*, volume 14, pages 1137–1145. Stanford, CA, 1995.

D. Pischel, J.H. Buchbinder, K. Sundmacher, I.N. Lavrik, and R.J. Flassig. Si: A guide to automated apoptosis detection, 2018. URL `https://doi.org/10.5281/zenodo.1220090`.

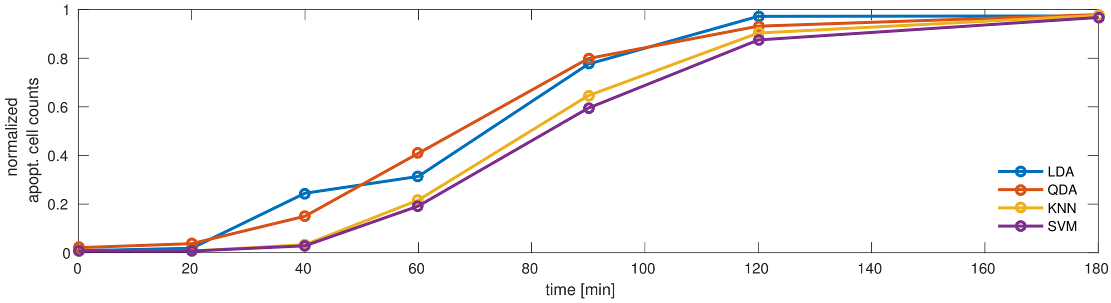

Figure 6: Model predictions on kinetics data.
